# Expression Status And Prognostic Value Of M6A-associated Genes in Gastric Cancer

**DOI:** 10.7150/jca.40866

**Published:** 2020-03-04

**Authors:** Kelei Guan, Xin Liu, Jianhao Li, Yanxia Ding, Juan Li, Guangying Cui, Xichun Cui, Ranran Sun

**Affiliations:** 1 Department of pharmacy, the First Affiliated Hospital of Zhengzhou University; 2 Precision Medicine Center, the First Affiliated Hospital of Zhengzhou University; 3 Key Laboratory of Clinical Medicine, the First Affiliated Hospital of Zhengzhou University

**Keywords:** gastric cancer, N6-methyladenosine, prognostic, WTAP, FTO.

## Abstract

**Purpose**: Gastric cancer (GC) is a primary cause of cancer-associated mortality worldwide. N6-methyladenosine (m6A) is one of the most common RNA modifications that involves in the progression of numerous cancers. However, the expression status and function of m6A-related genes in gastric cancer is still not well understood. The current study is aimed to investigate the expression status and determinate prognostic value of m6A-related genes in gastric cancer.

**Methods**: m6A-asssociated gene expression was evaluated via analyzing the expression data of GC patients from the Cancer Genome Atlas (TCGA) and Gene Expression Omnibus (GEO) database. The protein expression levels of m6A-associated molecules were further validated by immunohistochemical (IHC) staining data from GC tissue microarray (TMA) cohort and Human Protein Atlas (HPA) database. Kaplan-Meier analysis was performed to assess the prognostic value of m6A-associated genes in gastric cancer. Risk score model was established by lasso COX regression analysis and its prognostic predicted efficiency was assessed by the receiver-operator characteristic (ROC) curve. Cox regression analyses were used for exploring risk factors related to GC patient prognosis.

**Results**: Most of m6A-related genes were upregulated at both mRNA and protein levels in gastric cancer tissues compared with that in normal gastric tissues. The expression levels of m6A-related genes were associated with clinicopathological features including race, age and TNM stage. High expression of WTAP and FTO predicted poor prognosis of GC patients. Survival analysis demonstrated that patients with high-risk scores had worse overall survival (OS) and ROC curves suggested the prediction performance for gastric patients. Moreover, Cox regression analyses indicated that m6A risk model score was a prognostic factor for OS and FTO upregulation might be a potential independent prognostic factor for recurrence-free survival (RFS) in gastric cancer patients.

**Conclusion**: m6A-related genes were dysregulated in GC and were closely associated with prognosis of GC patients. FTO might serve as a novel prognostic biomarker for gastric cancer, while the m6A-related risk score might be informative for risk assessment and prognostic stratification.

## Introduction

Gastric cancer (GC) ranks fifth and third among all cancers in terms of incidence and mortality respectively worldwide in 2018 1, 2. The development and progression of GC is a complicated multistep process, including a plenty of genetic and epigenetic changes 3. During the past decades, various strategies have been made for GC treatment, and the early diagnosis and treatment of GC have been improved significantly 4. Though early stage GC patients can be cured and have a good prognosis, early diagnosis is very challenging 5. GC patients at late stage have poor prognosis and higher mortality due to the lack of efficient diagnosis at early stage 6. Therefore, developing an efficient and potent strategy for GC early diagnosis and treatment is urgently needed.

N6-methyladenosine (m6A) modification is the methylation of the adenosine base at the nitrogen-6 position of mRNA which was first discovered as an abundant nucleotide modification in eukaryotic messenger RNA in 1974^7^-^9^. M6A modifications is regulated by three types of enzymes: “writers” (methyltransferases, including WTAP, KIAA1429, RBM15/15B, and METTL3/14/16), “readers” (YTH domain containing RNA binding proteins and heterogeneous nuclear ribonucleoprotein, including YTHDF1/2/3, YTHDC1, HNRNPC and HNRNPA2B1) and “erasers” (demethylases, including ALKBH5 and FTO) 10-12.

Studies have shown that m6A modification has the character of dynamically reversible as same as the DNA and histone modifications 13. It plays a pivotal role in regulating precursor mRNA maturation, translation and degradation 14. In addition, m6A modification could also affect tissue development 15, cell self-renewal and differentiation, control of heat shock response 16, DNA damage response 17, circadian clock controlling and the development of multiple forms of human diseases, including cancer 7. Emerging evidence has demonstrated that m6A modification play a critical role in a great variety of human cancers 14, including breast cancer 18, 19, lung cancer 20, acute myeloid leukemia (AML) 21, 22, glioblastoma 23, hepatoblastoma 24, colorectal cancer 25 and so on 14. However, the function of m6A methylation in gastric cancer initiation, progression and prognosis is still not fully understood.

Herein, we first explored the expression pattern of m6A-related genes by bioinformatics analysis of TCGA and GEO database. The expression pattern was further confirmed by immunohistochemical (IHC) staining of GC tissue microarray cohort. The correlation between m6A-associated genes expression and clinicopathological features was analyzed and high expression of WTAP and FTO predicted poor prognosis of GC patients. Moreover, our results also demonstrated that the dysregulated expression of m6A-related genes could affect the overall survival (OS) and recurrence-free survival (RFS) of GC patients. Our findings suggest that FTO might serve as a novel prognostic biomarker for gastric cancer, while the m6A-related risk score might be informative for risk assessment and prognostic stratification.

## Materials and Methods

### Expression data sets download and bioinformatics analysis

The TCGA-GC cohort data of 32 normal patients and 368 GC patients and all relevant clinical date were downloaded from The Cancer Genome Atlas (TCGA, https://tcga-data.nci.nih.gov/tcga/). 6 sets of independent microarrays, including GSE112369, GSE26899, GSE79973, GSE103236, GSE55696, GSE15459, were extracted from the Gene Expression Omnibus (GEO, https://www.ncbi.nlm.nih.gov/geo/) database. The characteristics of 6 microarrys, including accession number, RNA-Seq platform, number of samples, country and publication year, were collected in **Table 1**. The expression profiles of m6A-related genes were analyzed by these datasets and the clinical prognosis of GC patient were evaluated through these datasets. The downloaded raw data pre-procession and bioinformatics analysis were conducted using the R studio software (3.51) as previous described 26.

### TMA cohorts

The tissue microarray (TMA), containing 20 gastric cancer specimens and 20 corresponding normal gastric tissue specimens, were acquired from April 2016 to December 2016 at the First Affiliated Hospital of Zhengzhou University. 4 paired gastric cancer tissues and adjacent normal tissues were obtained at December 2019. All the patients did not receive any immunotherapy, chemotherapy and radio therapy before surgery. This study was approved by The Institutional Review Board of the First Affiliated Hospital of Zhengzhou University and all the patients signed informed consent. In addition, we further verified the protein expression of m6A-related molecules through analyzing another GC TMA cohort from the Human Protein Atlas (HPA, https://www.proteinatlas.org/) database. The clinicopathological features of 20 gastric cancer patients were described in **Supplementary Table 1**.

### Immunohistochemical (IHC) staining and Western blot

TMA sections (5 μm thick) were deparaffinized and hydrated. 0.3% hydrogen peroxide was used to block endogenous peroxidases activity and antigen retrieval. TMA sections were incubated with primary antibody overnight at 4°C after blocking for one hour at room temperature. Then, TMA sections were incubated with secondary biotinylated goat anti-Rabbit antibody, and then detected by SignalStain® DAB (Cell Signaling Technology, Danvers, MA) and counterstained with haematoxylin QS (Vector Laboratories). The IHC staining results were evaluated independently by two pathologists who were blinded to the clinicopathologic data. According to the proportion of positive cells, samples were scored as follows: 0+, none; 1+, <25%; 2+, 25%-50%; 3+, 51%-75%; and 4+, 75%-100%. The staining intensity was evaluated as follows: 0, none; 1, weak; 2, medium; and 3, strong. The final score (range 0-12) was calculated by multiplying the two sub-scores. Samples were classified as low expression (0-3), moderate expression (4-6) and high expression (9-12) respectively. Western blot was performed as the following procedure. Briefly, the protein of fresh tissues was acquired by using protein extraction reagent (Beyotime, Beijing, China) with a protease inhibitor cocktail (Roche, Indianapolis, IN, USA). Same amount of protein was electrophoresed on 10% SDS-PAGE gels and afterwards transferred to polyvinylidene difluoride membranes (Millipore, Billerica, MA, USA). Phosphate buffer saline (PBS) containing 5% skim milk was used for blocking the membranes for 1 hour. Then the membrances were incubated with primary antibody overnight at 4°C. After secondary antibody (Santa Cruz Biotechnology, Santa Cruz, CA, USA) incubation for 1 hours, the membranes were used to expose the photographic film. All antibodies used in our study were described in **Supplementary Table 2**.

### Statistical analysis

SPSS software (version 23.0, Inc., Chicago, IL) and GraphPad Prism 7 Software (GraphPad Software, Inc, La Jolla, CA, USA) were used for statistical analysis. The Student's t test (unpaired, two-tailed) was used for analyzing the difference between two independent groups. The TMA analysis was accomplished by the Fisher's exact test. We obtained the best cut-off value for each gene and its survival curves through RStudio (0.99.447). Overall survival (OS) analysis and recurrence free survival (RFS) analysis were finished through the Kaplan-Meier method. The prognostic model was performed by RStudio and ROC curve was applied to confirm prognostic efficiency. In addition, the independent factors were sought through Cox regression analysis of univariate and multivariate. In all cases, “P<0.05” was considered as statistically significant.

## Results

### Bioinformatics analysis of the expression pattern of m6A-associated genes in gastric cancer

**Figure 1:**
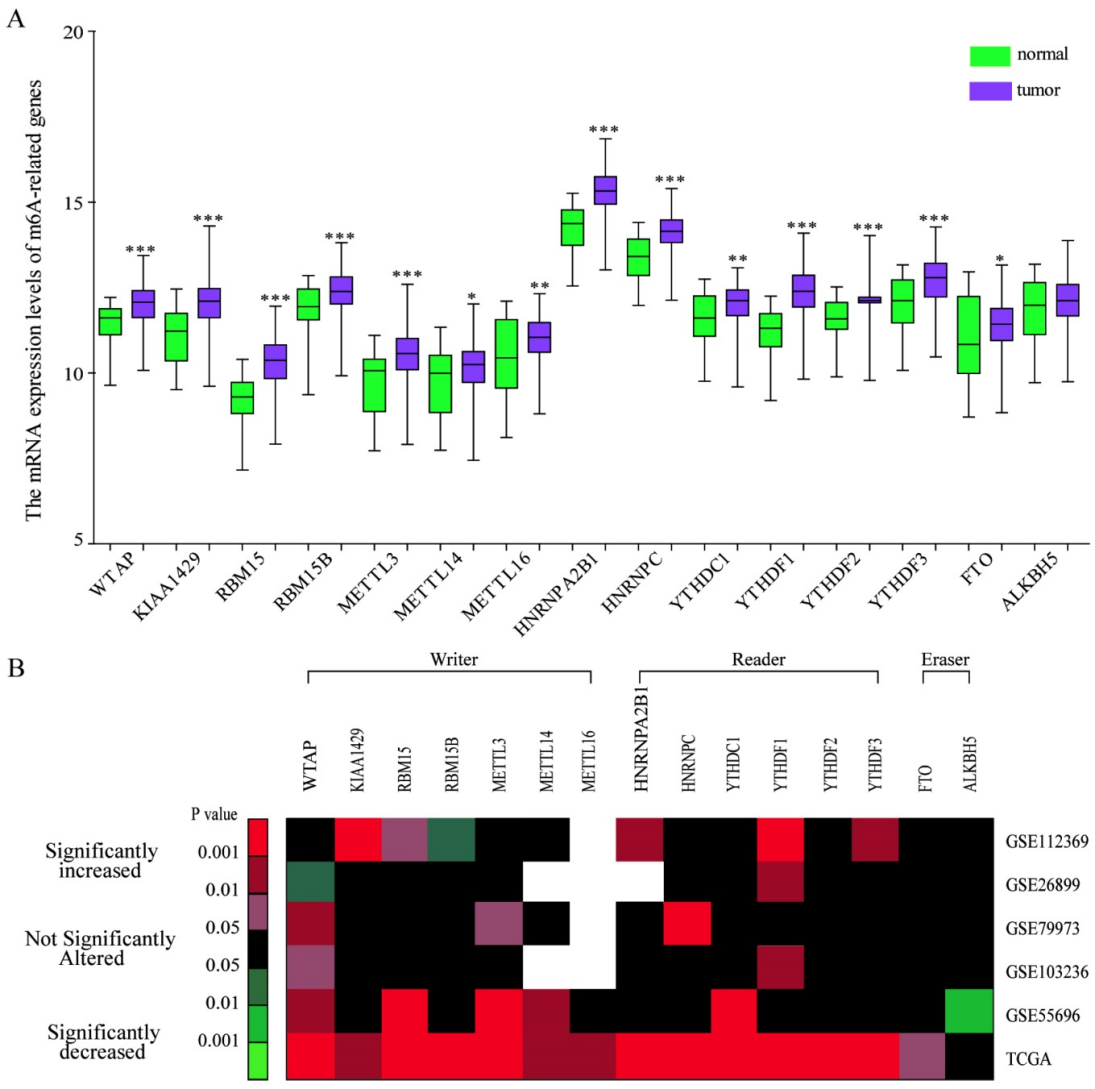
** Bioinformatics analysis of the expression pattern of m6A-associated genes in human GC tissues.** (A) Bioinformatics analysis of the mRNA expression pattern of m6A-associated genes in GC and normal tissues based on the data from TCGA-GC cohort. (B) Heatmap exhibiting the mRNA expression alteration of m6A-associated genes in five independent GEO microarray datasets. Red indicates up-regulated; green indicates down-regulated; black indicates not significant; blank indicates genes are not expressed or absent in the datasets. Statistical analysis was performed in Student's t test (unpaired, two-tailed).

To explore the expression pattern of m6A-related genes in human GC, we first extracted and analyzed the expression data of m6A-related genes from the Cancer Genome Atlas (TCGA) database. As shown in Figure 1A, the expression levels of all the known m6A-related genes including “writer”, “reader'', and “eraser” were upregulated in GC tissues compared with that in normal gastric tissues except ALKBH5. We further verified the expression pattern of m6A-related genes in 5 independent GC GEO datasets with microarray platforms and GEO datasets analysis showed a similar expression pattern of m6A-related genes (Figure 1B). Together, the results showed that the expression pattern of most m6A-associated genes was upregulated in comparison with that in normal gastric tissues.

### IHC analysis of the protein expression of m6A-related molecules in gastric cancer

**Figure 2:**
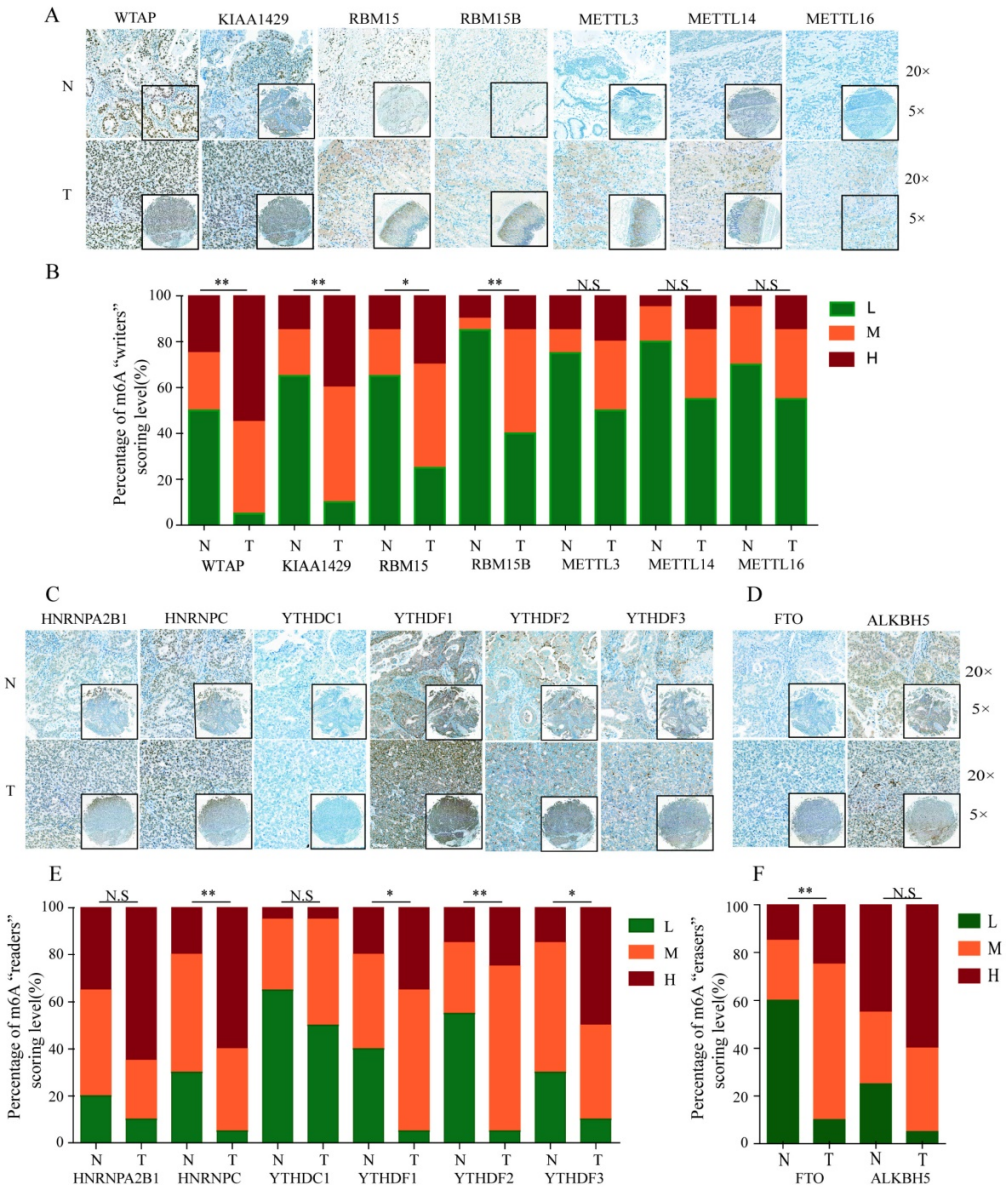
** IHC analysis of the protein expression of m6A-related molecules in GC and normal tissues.** (A) Representative IHC staining of m^6^A-related “writers” in GC and normal tissues in TMA cohort. (B) Comparison of the relative expression of m^6^A-related “writers” between GC and normal tissues in TMA cohort. (C) Representative IHC staining of m^6^A-related “readers” in GC and normal tissues in TMA cohort. (E) Comparison of the relative expression of m^6^A-related “readers” between GC and normal tissues in TMA cohort. (D) Representative IHC staining of m^6^A-related “erasers” in GC and normal tissues in TMA cohort. (F) Comparison of the relative expression of m^6^A-related “erasers” between GC and normal tissues in TMA cohort. (N-normal, T-tumor, *P<0.05, **P<0.01, ***P<0.001, N.S: no significance).

**Figure 3:**
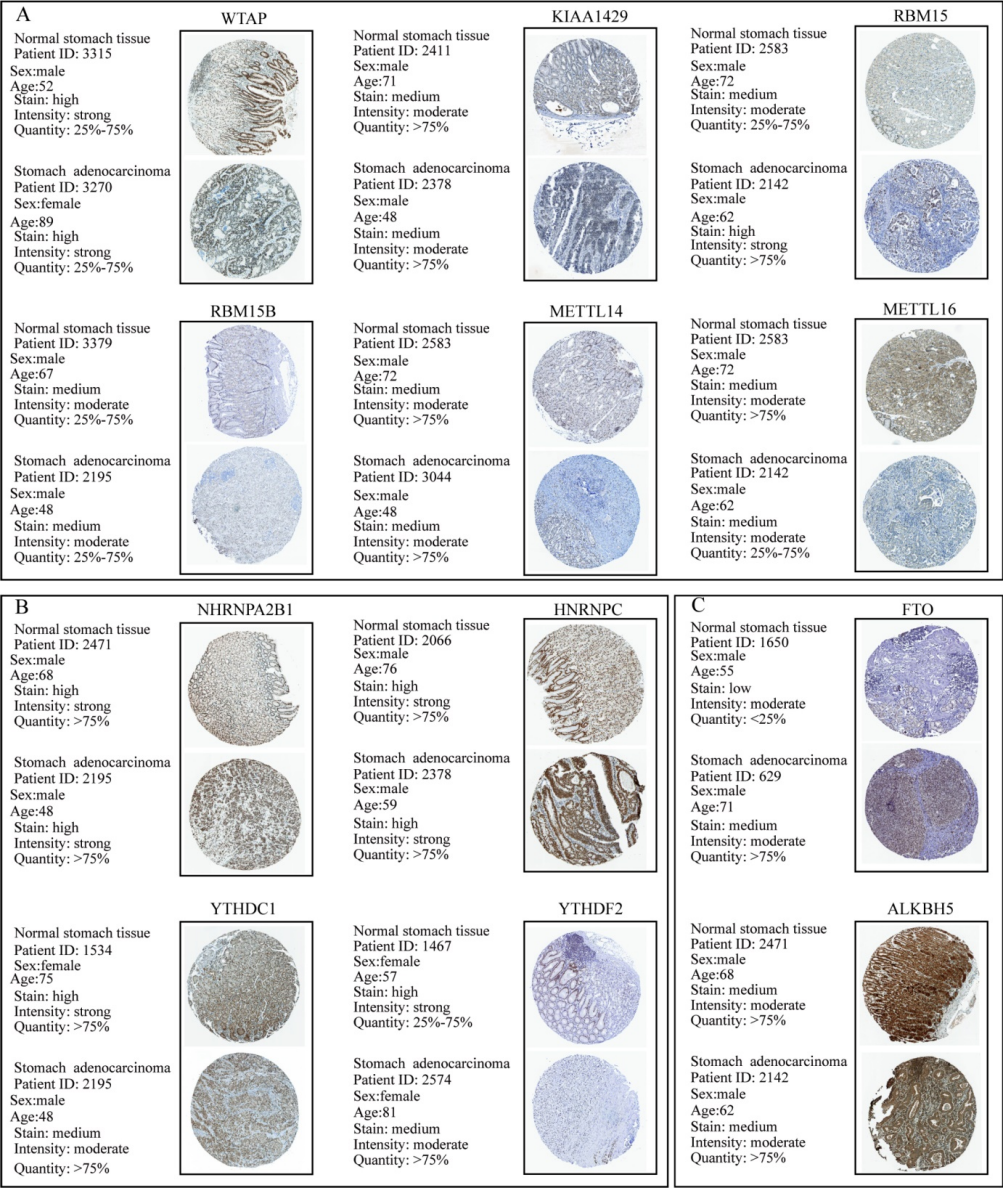
** IHC analysis of the protein expression of m6A-related molecules in The Human Protein Atlas database**. (A) Information and representative IHC staining of m6A-related “writers” in The Human Protein Atlas database. (B) Information and representative IHC staining of m6A-related “readers” in The Human Protein Atlas database. (C) Information and representative IHC staining of m6A-related “erasers” in The Human Protein Atlas database.

To further validate the expression pattern of m6A-related molecules in GC specimens and normal gastric tissues, we performed immunohistochemical staining analysis of GC TMA from the ZZU cohort and the Human Protein Atlas (HPA) database. IHC staining analysis of m6A “writer” suggested that WTAP, KIAA1429 and RBM15/15B expression were upregulated in GC tumor tissues compared with that in normal gastric tissues at protein level, which was consistent with the expression pattern of genes. However, the protein levels of METTL3/14/16 had no significant changes between GC and normal gastric tissues (**Figure 2A** and** 2B**). Of m6A “reader”, HNRNPC and YTHDF1/2/3 were overexpressed at protein level, while HNRNPA2B1 and YTHDC1 had no difference (**Figure 2C** and** 2E**). In terms of m6A “eraser”, IHC staining analysis demonstrated that FTO had a significant increase at protein expression levels, but not for ALKBH5 (**Figure 2D** and **2F)**. The similar results were obtained by analyzing IHC staining data from the HPA database (**Figure 3**). Furthermore, we also investigated the protein expression levels of m6A-related genes in fresh tumor tissues and the results indicated that the expression level of WTAP and FTO were markedly higher in GC tissues compared with normal control tissues (**Supplementary Figure 1**).

### Relationship between the expression of m6A-associated genes and clinicopathological features in GC

**Figure 4:**
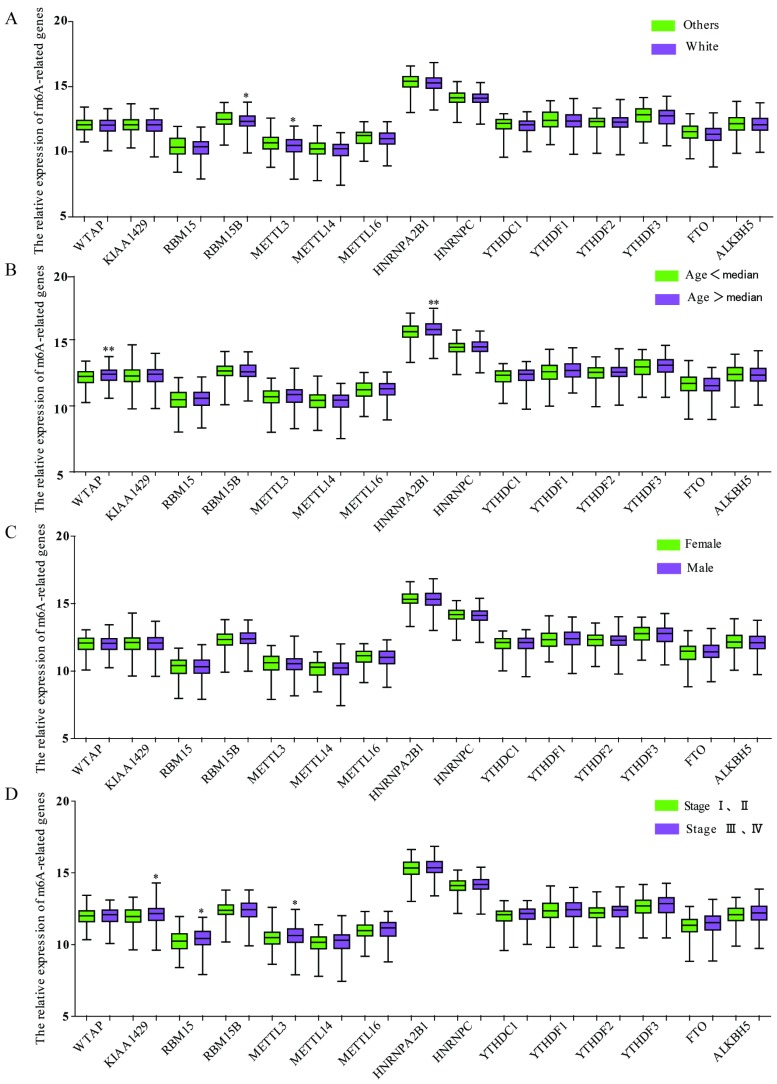
** The relationship between m6A-associated genes expression and clinicopathological features in GC.** (A)The relationship between the expression of m6A-associated genes and race was analyzed. (B) The relationship between the expression of m6A-associated genes and age was analyzed. (C) The relationship between the expression of m6A-associated genes and sex was analyzed. (F) The relationship between the expression of m6A-associated genes and TNM stage was analyzed. *P<0.05.

We also investigated the relationship between the expression of m6A-associated genes and clinicopathological features in GC. As shown in **Figure 4A**, race was related to the expression of RBM15B and METTL3, and the White had a significant downregulated expression of RMB15B and METTL3. The expression of WTAP or HNRNPA2B1 was associated with age, while the group with age over median had a significant upregulated expression of WTAP and HNRNPA2B1 (**Figure 4B**). There was no significant correlation between expression of m6A-related genes and genders (**Figure 4C**). Intriguingly, TNM stage was associated with the expression of KIAA1429, RBM15 and METTL3 and the group with TNM stage III and IV had a significant enhanced expression (**Figure 4D**).

### Relationship between the expression of m6A-associated genes and prognosis in GC

**Figure 5:**
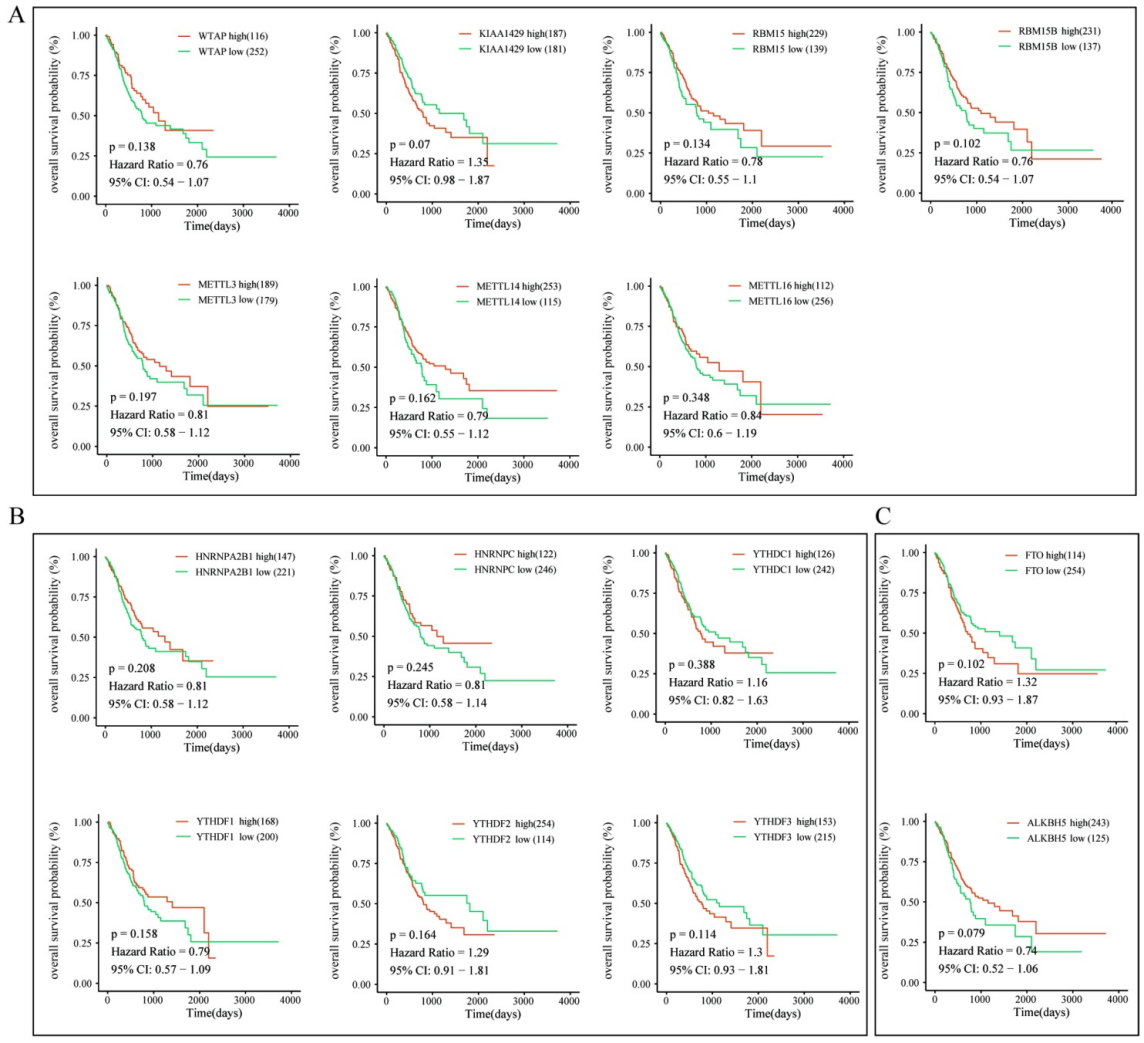
** The correlation between the expression levels of m6A-related genes and overall survival (OS) rates in GC patients.** (A) The correlation between the expression levels of m6A “writers” and OS rates in GC patients. (B) The correlation between the expression levels of m6A “readers” and OS rates in GC patients. (C) The correlation between the expression levels of m6A “erasers” and OS rates in GC patients. (red: high expression; green: low expression).

**Figure 6:**
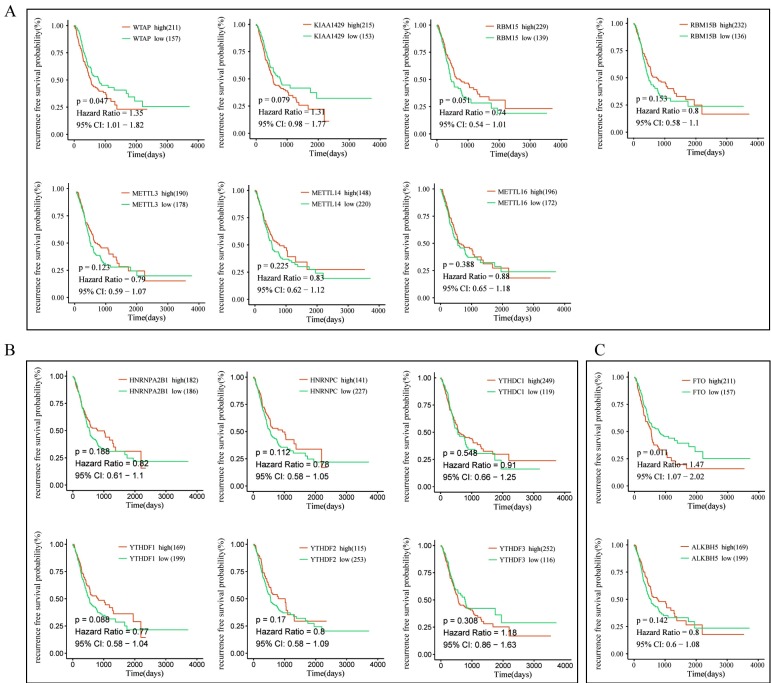
** The correlation between the expression levels of m6A-related genes and relapse free survival (RFS) rates in GC patients.** (A) The correlation between the expression levels of m6A “writers” and RFS rates in GC patients. (B) The correlation between the expression levels of m6A “readers” and RFS rates in GC patients. (C) The correlation between the expression levels of m6A “erasers” and RFS rates in GC patients. (red: high expression; green: low expression).

To further explore the prognostic role of m6A-associated genes in GC, we analyzed the correlation of m6A-related gene expression with corresponding clinical follow-up information through Kaplan-Meier analysis. GC patients were classified into the high expression group and the low expression group according to the best cut-off value. The results revealed that the expression levels of m6A-related genes were not significantly related with overall survival (OS) in GC (**Figure 5A-5C**). However, high levels of WTAP or FTO predicted poor recurrence-free survival (RFS) rates in GC patients (**Figure 6A-6C**).

### Prognostic predictor for GC patients

**Figure 7:**
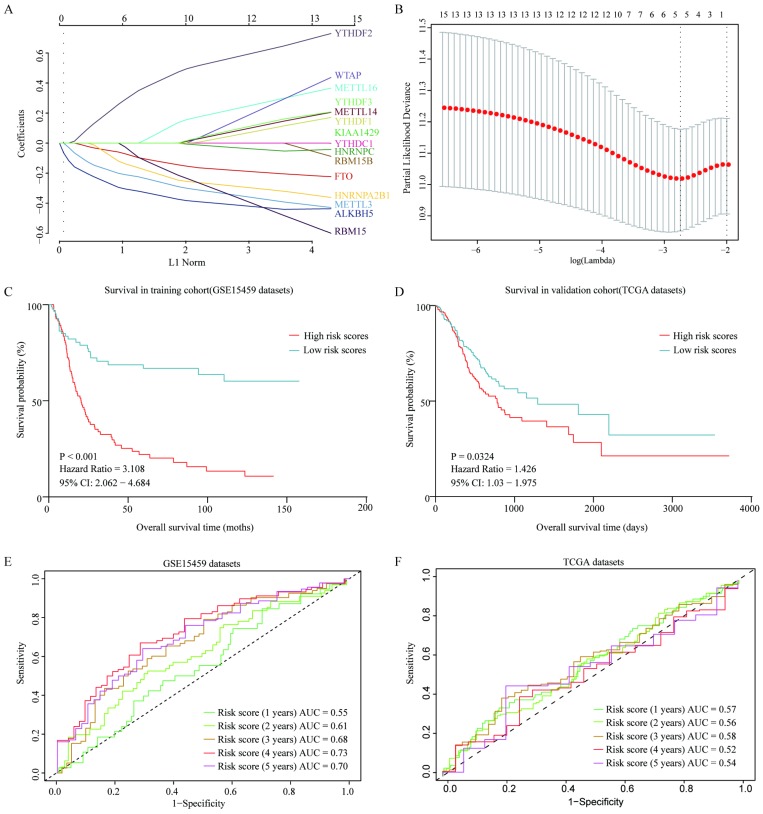
** Construction and verification of a survival risk score model as prognostic predictor for gastric patients.** (A) Ten-time cross-validation for tuning parameter selection in the LASSO model. (B) LASSO coefficient profiles of 15 prognostic genes. (C) Survival analysis in GSE15459 cohort. (D) Survival analysis in TCGA cohort. (E) ROC curves in GSE15459 cohort. (F) ROC curves in TCGA cohort.

To further evaluate the prognostic value of m6A-associated genes, we established a survival risk score model by Lasso Cox regression as follow: risk score = -0.2628 × Expression of ALKBH5 - 0.04741× Expression of FTO - 0.07108 × Expression of HNRNPA2B1 - 0.17652 × Expression of METTL3 + 0.19988 × Expression of YTHDF2 (**Figure 7A** and** 7B**). GC Patients were divided into two groups by a median of risk score. High risk score of patients had a significant shorter overall survival than that in patients with low risk score (*P*<0.001) in both GSE15459 and TCGA cohort (**Figure 7C** and **7D**). Moreover, the performance of this prognostic predictor was confirmed by ROC curve analysis. As shown in **Figure 7E,** the area under the ROC curve (AUC) were 0.55, 0.61,0.68, 0.73 and 0.70 in the 1-, 2-, 3-, 4- and 5-year, respectively in GSE15459 cohort, which indicated its potential to predict prognosis. The ROC curve analysis results were consistent in TCGA cohort (**Figure 7F**). Thus, the risk score model showed some predicting power.

### Risk factor analysis of m6A-associated genes in GC

**Figure 8:**
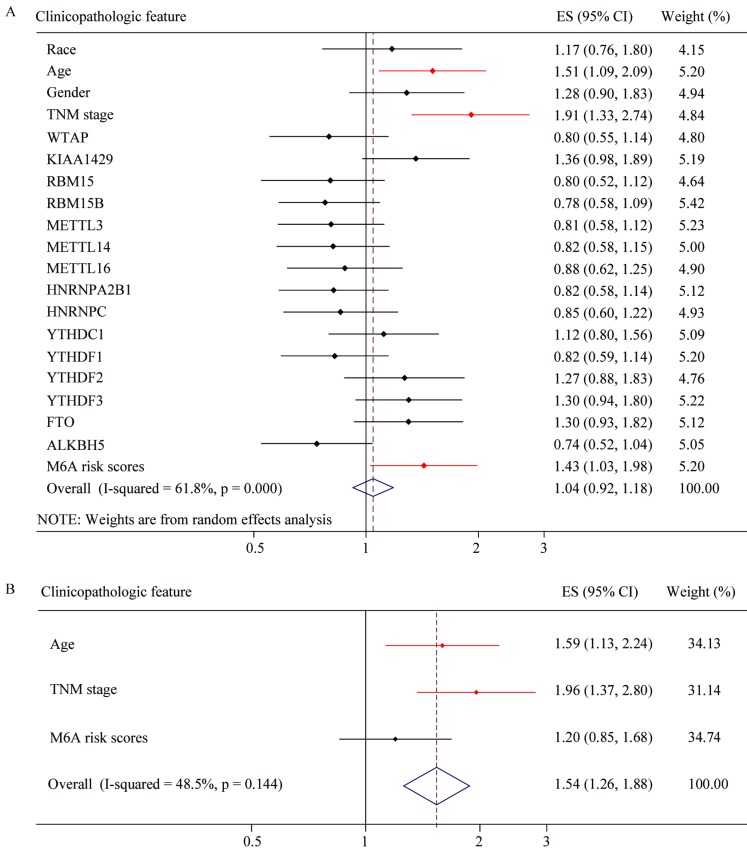

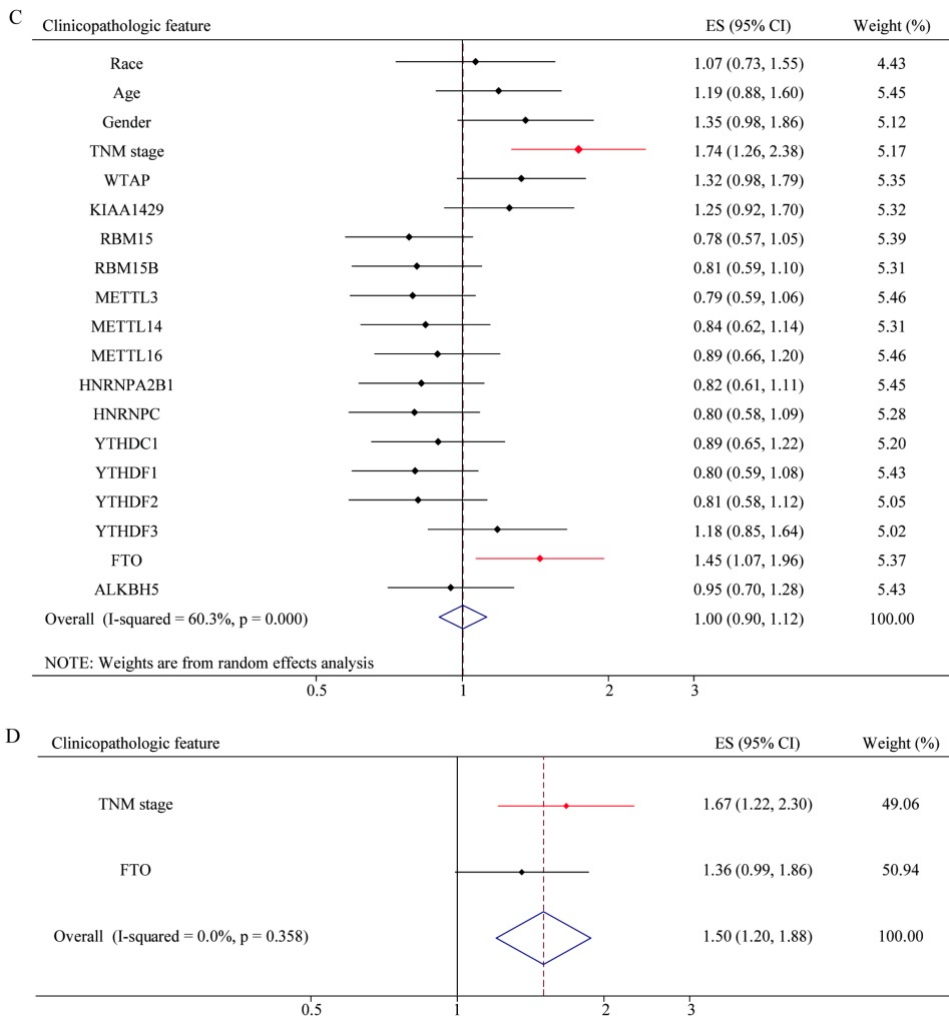
** Univariate and multivariate Cox regression analyses of the TCGA database.** (A) Univariate Cox regression analysis of the OS of GC patients. (B) Multivariate Cox regression analysis of the OS of GC patients. (C) Univariate Cox regression analysis of the RFS of GC patients. (D) Multivariate Cox regression analysis of the RFS of GC patients.

To explore whether risk factor was associated with GC patient prognosis, we performed univariate and multivariate Cox regression. The results of univariate Cox regression revealed that m6A risk score was significant risk factor for OS (**Figure 8A**). TNM (tumor, node, and metastasis) stage was significant risk factor for OS and RFS (**Figure 8A** and **8C, Table 2** and **3**). High expression of FTO was significant risk factor for RFS (**Figure 8C**). The results of multivariate Cox regression indicated that TNM stage was an independent risk factor for OS and RFS (**Figure 8B** and **8D, Table 2** and **3**), and FTO overexpression (HR=1.356, *P*=0.057, 95%CI: 0.991-1.857) might be an independent risk factor for RFS (**Figure 8D**).The results of Univariate and Multivariate Cox regression indicated that age might be a confounder factor (**Table 2**). Further, stratified analysis suggested that TNM stage and RBM15B were independent risk factors for OS in patients with age≤median, and TNM stage also was a risk factor factors for OS in patients with age >median (**Supplementary table 3** and** 4**). Furthermore, we conducted hallmark pathway, KEGG and GO analysis via GSVA. The result suggested that m6A risk score related with gene signature was involved in multiple signaling pathways (**Supplementary Figure 2**).

## Discussion

N6-methyladenosine (m6A) modification is the most common modification in human mRNA 27, and it is considered as a new layer of epigenetic regulation on mRNA processing, translation 28 and stability 8, 29. Mounting evidence has proved that dysregulation of m6A modification is closely associated with various human physiological and pathological phenomena, including obesity, immuno-dysregulation, carcinogenesis^30^, ^31^ and so on 32, 33. As the development of the m6A-sequencing (m6A-seq) technology, it has been achieved to explore the roles of m6A mRNA modification in cancer biology 34, 35. In recent years, emerging studies have suggested that m6A-related genes have crucial roles in the initiation and progression of cancers. For example, it has been reported that METTL3 not only promotes the growth and tumorigenesis of acute myeloid leukaemia cells but also suppresses renal cell carcinoma 22, 36. METTL14 has been found to suppress the metastatic potential of hepatocellular carcinoma and increase the tumorigenesis of glioblastoma stem cells 11, 30. However, the function of m6A-related genes in gastric cancer initiation and progression are not fully known yet. In this study, we first explored the roles of m6A-related genes in the regulation of GC.

The analysis of TCGA and GEO databases showed that the expression levels of most m6A-related genes were upregulated in GC, including WTAP, KIAA1429, RBM15/5B, METTL3/14/16, HNRNPA2B1, HNRNPC, YTHDC1, YTHDF1/2/3 and FTO^37^. In addition, we also explored the protein expression of m6A-associated molecules in GC tissues through an IHC analysis and found that many m6A-related molecules expression was upregulated at protein level, including WTAP, KIAA1429, RBM15/15B, HNRNPC, YTHDF1/2/3 and FTO. The protein expression was consistent with gene expression level. These findings suggested that m6A-related genes were dysregulated in GC tissues and some of them might have an oncogenic role in GC patients. Consistent with the published results, Jasmin P *et al*. found that YTHDF2 was not only overexpressed but also essential for disease initiation and progression in human acute myelocytic leukemia (AML)^21^. Zhao *et al*. reported that YTHDF1 was marked upregulated in hepatocellular carcinoma (HCC) and played a crucial role in regulating HCC cell cycle progression and metabolism^38^. In addition, FTO was found highly expressed in lung squamous cell carcinoma (LUSC) and knockdown FTO suppressed cancel cell viability and invasion^20^. Xiaoyu C *et al*. revealed that the expression level of KIAA1429 in liver tumor was significantly higher than that in normal liver tissues and high expression of KIAA1429 was significantly related with poor overall survival^39^. Furthermore, it was reported that bladder cancer patients with positive WTAP expression had higher post-operative recurrence compared with those with negative WTAP expression^40^. Our results suggested that m6A-associated genes had a crucial role in initiation and progression of GC. The significant relationship between the expression of some m6A-associated genes and clinicopathological features had been confirmed in GC. For example, down-regulated expression of RBM15B and METTL3 was associated with the race. The age could influence the expression of WTAP and HNRNPA2B1. KIAA1429, RBM15 and METTL3 in GC cohorts with stage III and IV were upregulated compared with that in GC cohorts with stage I and II. Our findings for the first time demonstrated the interrelation of the expression of m6A-related genes and clinicopathological feature and raised the new direction for further research.

Moreover, we found that the upregulated expression of WATP and FTO were significant associated with poor prognosis in GC patients through the Kaplan-Meier method analysis of TCGA datasets. GC patients with high expression of WTAP and FTO had poor recurrence-free survival rates. Numerous studies had demonstrated that the dysregulation of m6A-associated genes were in connection with poor prognosis. For instance, Niu Y *et al*. found that FTO was overexpressed and significantly related with lower survival rates in patients with breast cancer^41^. FTO might be regarded as a novel target for breast cancer therapy. Li Y *et al*. found that aberrant expression of FTO as demethylase genes had a significant prognostic value in gastric cancer patients, suggesting that FTO might have a crucial role in GC progression and metastasis^42^. In addition, a large amount of studies suggested that WTAP as a novel oncogenic protein played a vital role in AML, and it also was a crucial factor on poor prognosis of malignant glioma^43^. Based on gene expression profile, we established a risk score model via lasso COX regression that had some predicted performance for prognosis of GC patients^44^, ^45^. The result of m6A risk score model suggested that m6A-related genes were significantly associated with prognosis of GC patients. Univariate analysis suggested that TNM stage, high expression of FTO and m6A risk score were prognosis factors in GC patients. Multivariate analysis indicated that TNM stage was independent prognostic factors. High expression of FTO (HR=1.356, *P*=0.057, 95%CI: 0.991-1.857) might be an independent prognostic factor in GC patients.

However, our study has some limitations. Though m6A-related genes have been demonstrated having high prognostic values in GC patients, their accurate mechanism in GC progression and prognosis need to be further studied. Moreover, the molecular mechanism by which m6A-associated genes facilitate GC development should be further explored both *in vivo* and *in vitro*.

## Conclusions

The m6A-associated genes were dysregulated in GC and played a crucial role in progression and prognosis of GC patients. This study not only suggests the potential value of m6A-related genes as novel prognostic biomarkers in GC but also offers a new direction for the diagnosis and treatment of gastric cancer.

## Supplementary Material

Supplementary figures and tables.

## Figures and Tables

**Table 1:** GEO microarray data enrolled to identify altered m6A targets in Gastric cancer.

Accession number	Platform	Number of samples		Country	Years
		Non-tumor	Gastric cancer		
GSE112369	Affymetrix	26	36	Japan	2018
GSE26899	Illumina	12	96	USA	2016
GSE79973	Affymetrix	10	10	China	2016
GSE103236	Agilent	9	10	Romania	2017
GSE55696	Agilent	19	58	China	2017
GSE15459	Affymetrix		200	Switzerland	2009
Total		76	410		

**Table 2:** Independent prognostic factors for OS by univariate and multivariate analysis in TCGA cohorts

Risk factors	Clinicopathological features	Univariate analysis			Multivariate analysis		
		HR	95% (CI)	*P* value	HR	95% (CI)	*P* value
Race	White	1.000	0.764-1.802	0.465			
	Others	1.173					
Age(years)	≤median	1.000	1.085-2.093	0.014*	1.000	1.123-2.217	0.009**
	>median	1.507			1.578		
Gender	Women	1.000	0.904-1.828	0.162			
	Men	1.285					
TNM stage	Stage I and II	1.000	1.328-2.736	0.000***	1.000	1.372-2.812	p<0.001***
	Stage III and IV	1.914			1.964		

**Table 3:** Independent prognostic factors for RFS by univariate and multivariate analysis in TCGA cohorts

Risk factors	Clinicopathological features	Univariate analysis			Multivariate analysis		
		HR	95% (CI)	*P* value	HR	95% (CI)	*P* value
Race	White	1.000	0.731-1.551	0.744			
	Others	1.065					
Age(year)	≤median	1.000	0.884-1.600	0.252			
	>median	1.189					
Gender	Women	1.000	0.980-1.861	0.067			
	Men	1.350					
TNM stage	Stage I and II	1.000	1.264-2.385	0.001**	1.000	1.215-2.304	0.002**
	Stage III and IV	1.736			1.673		
FTO	Low	1.000	1.068-1.955	0.017*	1.000	0.991-1.857	0.057
	High	1.445			1.356		

## Abbreviations

m6A: N6-methyladenosine
GC: gastric cancer
TMA: tissue microarray
TCGA: The Cancer Genome Atlas
GEO: Gene Expression Omnibus
HPA: Human Protein Atlas database
OS: overall survival
RFS: recurrence-free survival
TNM: tumor node metastasis
AML: acute myeloid leukaemia
FTO: Fat Mass and Obesity associated protein
WTAP: wilms' tumour 1-associated protein
ALKBH5: alkB homolog 5
METTL3: Methyltransferase like 3
METTL14: Methyltransferase like 14
METTL16: Methyltransferase like 16
HNRNPA2B1: Heterogeneous Nuclear Ribonucleoprotein A2/B1
HNRNPC: Heterogeneous nuclear ribonucleoprotein C (C1/C2)
RBM15: RNA binding motif protein 15
RBM15B: RNA binding motif protein 15B
YTHDC1: YTH domain containing 1
YTHDF1: YTH N6-methyladenosine RNA binding protein 1
YTHDF2: YTH N6-methyladenosine RNA binding protein 2
YTHDF3: YTHN6-methyladenosine RNA binding protein 3
