# Supplementary material for: Expression Status And Prognostic Value Of M6A-associated Genes in Gastric Cancer
Source: J Cancer. 2020 Mar 4;11(10):3027–40. doi: 10.7150/jca.40866 (PMC7086255; doi:10.7150/jca.40866)
Supplement: Supplementary file 1 — Supplementary figures and tables. [file jcav11p3027s1.pdf]

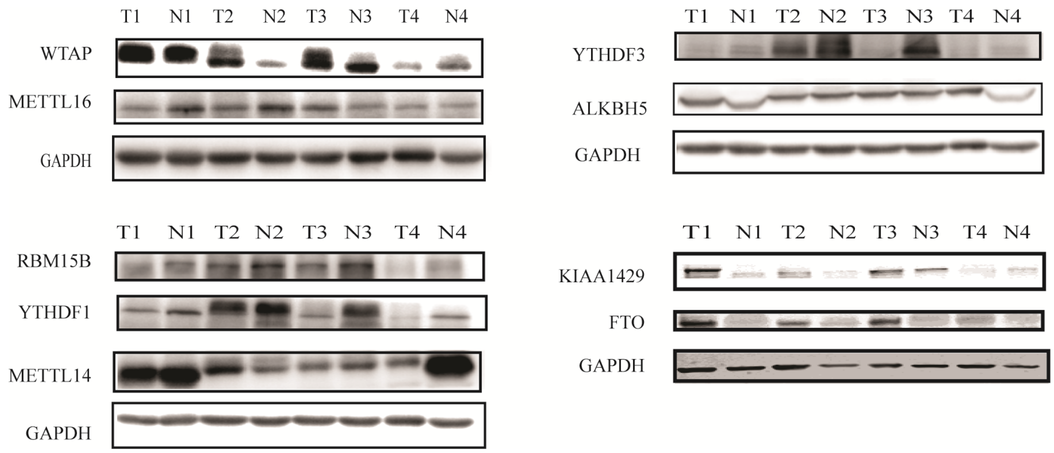

Supplementary Figure 1. Protein expression of m6A-related molecules in 4paired gastric tumor tissues (T) and normal control tissues (N)

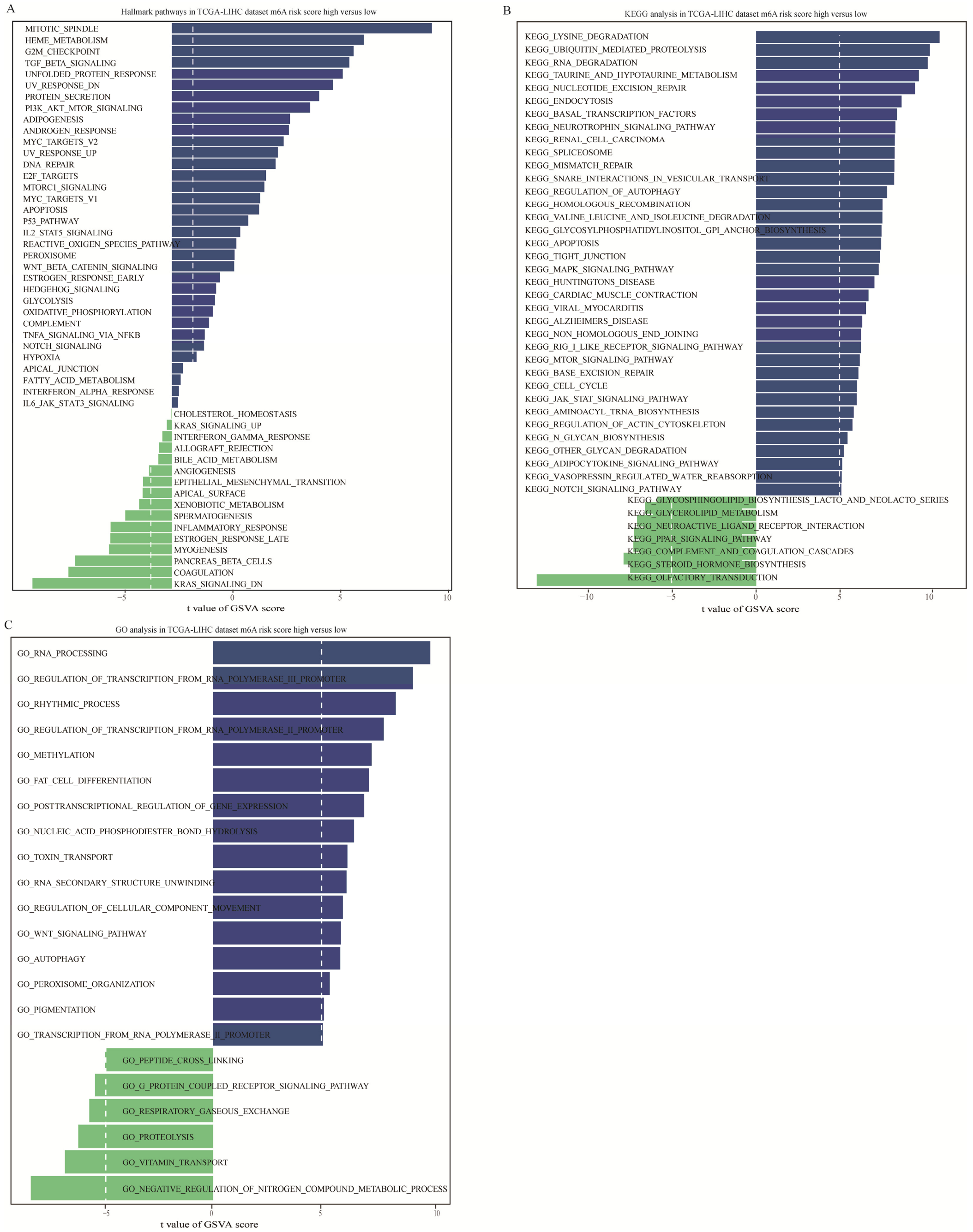

Supplementary Figure 2. M6A risk score related with gene signature involved signaling pathways.

Supplementary Table 1. The clinicopathological features of 20 GC patients.

Sample	Race	Age	Gender	Grade	TNM stage
1	Han	64	Male	2	IIIa
2	Han	64	Male	2	IIa
3	Han	54	Male	3	IIb
4	Han	71	Male	2	IIIa
5	Han	65	Male	2	Ib
6	Han	68	Male	2	IIIb
7	Han	71	Male	2	IIIa
8	Han	73	Male	2	IIa
9	Han	58	Female	3	IIIc
10	Han	59	Female	2	IIb
11	Han	70	Male	3	IIb
12	Han	65	Male	2	IIIc
13	Han	59	Male	2	IIb
14	Han	67	Male	2	IIIa
15	Han	48	Female	3	IIIb
16	Han	65	Male	1	IIa
17	Han	59	Male	3	IIb
18	Han	64	Male	2	Ib
19	Han	53	Male	3	IIIa
20	Han	48	Female	3	IIIa

Supplementary Table 2. The information of antibodies used in this study.

	Antibody	IHC	WB	Specificity	Company
**M6A writer**	WTAP	1:500	1:1000	Mouse Monoclonal	Proteintech Group, China
	KIAA1429	1:200	1:1000	Rabbit Polyclonal	Proteintech Group, China
	RBM15	1:500	1:1000	Rabbit Polyclonal	Proteintech Group, China
	RBM15B	1:200	1:1000	Rabbit Polyclonal	Proteintech Group, China
	METTL3	1:200	1:1000	Rabbit Polyclonal	Proteintech Group, China
	METTL14	1:200	1:1000	Rabbit Polyclonal	Proteintech Group, China
	METTL16	1:200	1:1000	Rabbit Polyclonal	Proteintech Group, China
**M6A eraser**	ALKBH5	1:500	1:1000	Rabbit Polyclonal	Proteintech Group, China
	FTO	1:200	1:1000	Rabbit Polyclonal	Proteintech Group, China
**M6A reader**	YTHDC1	1:200	1:1000	Rabbit Polyclonal	Proteintech Group, China
	YTHDF1	1:200	1:1000	Rabbit Polyclonal	Proteintech Group, China

	YTHDF2	1:200	1:1000	Rabbit Polyclonal	Proteintech Group, China
	YTHDF3	1:200	1:1000	Rabbit Polyclonal	Proteintech Group, China
	HNRNPA2B1	1:500	1:1000	Rabbit Polyclonal	Proteintech Group, China
	HNRNPC	1:200	1:1000	Rabbit Polyclonal	Proteintech Group, China

Supplementary Table3. Independent prognostic factors for OS by stratified analysis in TCGA cohorts with age ≤ median.

Risk factors	Clinicopathological features	Univariate analysis			Multivariate analysis		
		HR	95%（CI）	*P* value	HR	95%（CI）	*P* value
Race	White	1.000	0.911-4.452	0.084			
	Others	2.014					
Gender	Women	1.000	0.901-2.622	0.115			
	Men	1.537					
TNM stage	Stage I and II	1.000	1.028-3.037	0.039*	1.000	1.055-3.118	0.031*

	Stage III and IV	1.767			1.813		
RBM15B	Low	1.000	0.364-0.975	0.039*	1.000	0.353-0.978	0.041*
	High	0.596			0.587		

Supplementary Table4. Independent prognostic factors for OS by stratified analysis in TCGA cohorts with age >median.

Risk factors	Clinicopathological features	Univariate analysis			Multivariate analysis		
		HR	95%（CI）	*P* value	HR	95%（CI）	*P* value
Race	White	1.000	0.543-1.570	0.767			
	Others	0.923					
Gender	Women	1.000	0.684-1.761	0.700			
	Men	1.098					
TNM stage	Stage I and II	1.000	1.384-3.511	0.001***			
	Stage III and IV	2.176					
